# Microbiome Diversity in Vaginal Fluid and Sensitivity Patterns in Preterm Premature Rupture of Membrane Cases

**DOI:** 10.7759/cureus.20999

**Published:** 2022-01-06

**Authors:** Poonam A Ambalpady, Subhra Samantroy, Anamika Mishra, Jyochnamayi Panda, Dipti Pattnaik, Pramila Jena

**Affiliations:** 1 Obstetrics and Gynaecology, Kalinga Institute of Medical Sciences, Bhubaneswar, IND; 2 Microbiology, Kalinga Institute of Medical Sciences, Bhubaneswar, IND

**Keywords:** multidrug resistant organisms, premature preterm labor rupture of membranes, enterococcus faecalis, antimicrobial sensitivity pattern, preterm birth

## Abstract

Introduction: Preterm premature rupture of membranes (PPROM) is spontaneous rupture of the fetal membranes before 37 completed weeks and before the onset of labor. PPROM occurs in 3% of all pregnancies and is responsible for approximately one-third of all preterm deliveries. It leads to increase in perinatal morbidity and mortality.

Aim: The present study aimed to characterize the microbiome of vaginal fluid, which will be helpful in the selection of empiric antimicrobial therapy.

Materials and Method: A prospective observational study was conducted in the Department of Obstetrics and Gynaecology, Pradyumna Bal Memorial Hospital, Kalinga Institute of Medical Sciences (KIMS), Bhubaneswar during the period of October 2019 to June 2021 to characterize the microorganisms in the vaginal fluid and their antimicrobial sensitivity patterns found in antenatal women presenting with PPROM. A total of 160 antenatal women diagnosed with PPROM, gestational age between 28 weeks to 36 weeks and 6 days were included in the study. High vaginal swabs were collected for microbial culture and sensitivity.

Results: Out of 160 samples, the growth of organisms was observed in 134 (85.09%) samples. Out of them, 133 were monomicrobial, one was polymicrobial. Common isolated infections included *Enterococcus faecalis* (17.39%), followed by *Staphylococcus aureus* (14.29%), *Escherichia coli* (11.18%), and *Staphylococcus haemolyticus* (6.21%). Most of them were sensitive to ampicillin followed by linezolid and vancomycin. S. aureus was most sensitive to linezolid followed by gentamicin and vancomycin. Most isolates were multidrug-resistant.

Conclusion: The empirical antimicrobial treatment started for PPROM management should be based on the established changing microbiological pattern and sensitivities with due consideration of geographical and demographic variations.

## Introduction

Preterm delivery is one of the most challenging obstetric complications met by obstetricians worldwide. Preterm deliveries affect one in 10 births (11%) and an even greater percentage is seen in developing countries. Preterm premature rupture of membranes (PPROM) is a said concern when the rupture of fetal membranes occurs prior to 37 completed weeks of gestation and before the onset of labor and is responsible for 40% of all preterm births and is associated with a high perinatal mortality rate of 60-80% [1,2]. There is evidence that suggests that premature rupture of fetal membranes (PROM) is related to biochemical processes which include disruption of collagen within the extracellular matrix of the amnion and chorion along with the programmed cell death of cells present in the fetal membranes. PPROM occurs probably due to a variety of pathologies activating the aforementioned mechanisms and pathways prematurely.

The etiology of PPROM is multifactorial, including genital tract infection, polyhydramnios, antepartum bleeding, acute trauma, cigarette smoking, history of PPROM in a previous pregnancy, genetic polymorphism, etc. [3]. The cumulative evidence concerning the clinical risk factors, fetal membrane histology, and amniotic fluid microbiology show a strong association with the presence of infection leading to the PROM [2]. The microorganisms that ascend via the lower genital tract to the fetal membranes result in the production and release of proinflammatory chemokines and cytokines, which may weaken the fetal membranes that lead to PPROM. In the process of inflammation, there may be prostaglandin release which prompts cervical changes consequently in preterm delivery. The structural and architectural integrity of the fetal membranes is disrupted by the actions of these bacterial enzymes, as a result, the membrane ruptures.

In cases when PPROM presents before 34 weeks of gestation, conservative management is opted to ensure fetal lung maturity; provided there is no clinical evidence of any intra-amniotic infection, fetal distress, or placental abruption. Recommended management strategy includes the use of corticosteroids, tocolytics, and antibiotics.

The dearth of rapid diagnostic methods for validation of organisms associated, unavoidably leads to inappropriate use of antibiotics. In such scenarios, prescribing empirical broad-spectrum antibiotics is noted prior to confirmatory microbiological studies. The rampant and improper use of antibiotics has been one of the causes for increase in the resistance patterns in the pathogens. The lack of recent adequate microbiological studies prior to commencement of antibiotics in cases of PPROM creates the need to study the existing local microbiological pattern and their sensitivity pattern to the commonly used empirical antibiotics in order to recommend the first-line choice for empirical treatment. This will also help prolong the latency period and improve the fetal and maternal outcome as well as prevent wastage of resources and antibiotic abuse.

The objective of this study was to identify the current trend of common organisms isolated from the high vaginal swab culture collected at the time of presentation with PPROM. Also, to find the antimicrobial sensitivity patterns of the isolates identified in the culture growth.

## Materials and methods

It is a prospective observational study done at our tertiary care center in the Department of Obstetrics and Gynaecology of Pradyumna Bal Memorial Hospital, Kalinga Institute of Medical Sciences (KIMS), Bhubaneswar on attending outpatient (OPD) or labor room; for a period of 18 months from September 2019 to March 2021, after due permission from the Institutional Ethics Committee and Review Board and written informed consent from the patients.

The sample size was calculated to be 139 considering a confidence level of 95% and taking an incidence of 10% for PPROM according to a previous study [4]. This was increased to 160 after considering a no response error of 15%.

All antenatal women with gestational age between 28 weeks to 36 weeks and 6 days presenting to the OPD or labor room with complaints of frank leaking per vagina for less than 24 hours were enrolled for the study. The inclusion criteria were age between 20 and 45 years, singleton pregnancy with a live fetus with any type of fetal presentation and without any history of other known medical illness like hypertensive disorder, diabetes mellitus, chronic kidney diseases, connective tissue disorder, etc.

Exclusion criteria were antenatal women with gestational age less than 28 weeks and more than 37 weeks, multifetal gestation, intrauterine device (IUD), fetal anomaly, gestational diabetes, polyhydramnios, placenta previa and prior use of antibiotics within a period of 7 days prior to leaking per vagina, women presenting with recent sexual intercourse history, i.e., within 24 hours and those with history of digital examination elsewhere prior visiting to our hospital.

Once the patients were enrolled for the study, an informed consent was taken. A thorough history, general physical and obstetrical examination was done when the patient presented to the hospital. Diagnosis of PPROM was confirmed by sterile Cusco’s speculum examination and visualizing a pool of amniotic fluid in the posterior fornix of the vagina and noticing the fluid flowing out through the cervical os with or without Valsalva maneuver. High vaginal swabs were taken using the commercially available sterile swab sticks taking all necessary aseptic precautions. The swabs were collected from all the participating women of the study those who fulfilled the inclusion criteria.

Collected samples were sent to the microbiology diagnostic laboratory in our hospital, for the automated culture to identify the organisms followed by the susceptibility tests to antimicrobials. Each sample was also inoculated into blood agar and MacConkey agar as per the routine standard of protocol [5]. All the inoculated plates were incubated at 37°C for 24 hours. All specimens underwent Gram staining. All the microorganisms were identified according to the standard protocol. Following the identification of the pathogen, the antibiotic susceptibility test was performed for the commonly used antimicrobials.

Statistical analysis was done using the SPSS software data version 18. Categorical data was represented as proportion and analysis done using the chi-square or Fischer’s exact test and a p-value ≤0.05 was considered as statistically significant.

## Results

A total of 160 women fulfilling the inclusion criteria were considered in this study. Table 1 illustrates the sociodemographic characteristics of the studied population. The average age (mean ± standard deviation) of the women in our study was 28.67 ± 4.27 years (ranging from 21 to 45 years). Greater number of primigravida presented with PPROM in comparison to multigravida. Half of the participants (50.6%) belonged to the late preterm group (Table 1).

**Table 1:** Sociodemographic characteristics of the women

Sociodemographic characteristics	Numbers	Percentage
Age		
20-29	101	63.12
30-39	56	35.0
40-45	3	1.87
Gravida		
Primigravida	89	55.62
Multigravida	71	44.37
Gestational age		
Very preterm (28-31 weeks)	45	28.12
Moderately preterm (32-33 weeks)	34	21.25
Late preterm (34-36 weeks)	81	50.62
Socioeconomic status		
Low income	35	21.87
Upper middle	23	14.38
Lower middle	102	63.75

The high vaginal swab cultures from the samples collected in women presenting with PPROM reported 22 different types of pathogens, some of the subspecies of the organisms could not be identified using the automated culture done in our microbiology laboratory. The frequencies of these various organisms isolated from all the high vaginal swabs are mentioned in Table 2.

**Table 2:** Frequency of microorganisms isolated from high vaginal swab HVS: high vaginal swab

HVS growth	Frequency	Percentage
Enterococcus faecalis	28	17.39
Staphylococcus aureus	23	14.29
Escherichia coli	18	11.18
Staphylococcus haemolyticus	10	6.21
Candida albicans	9	5.59
*Klebsiella pneumoniae* ssp. pneumoniae	8	4.97
Acinetobacter baumannii	7	4.35
Enterococcus spp.	7	4.35
Pseudomonas aeruginosa	7	4.35
Staphylococcus epidermis	5	3.11
Acinetobacter spp.	2	1.24
Candida tropicalis	2	1.24
*Enterobacter cloacae* complex	2	1.24
Candida glabrata	1	0.62
Citrobacter koseri	1	0.62
Citrobacter spp.	1	0.62
Enterobacter spp.	1	0.62
Enterococcus faecium	1	0.62
Proteus mirabilis	1	0.62
Serratia marcescens	1	0.62
Staphylococcus saprophyticus	1	0.62
*Streptococcus agalactiae*—Group B	1	0.62
No growth	24	14.91
Total	161	100

One of the high vaginal swabs taken from a patient grew two different organisms, *Acinetobacter baumannii* and *Klebsiella pneumoniae* ssp. pneumoniae. Hence, a total of 161 organisms are reported against the sample of 160 patients. No growth or sterile culture reports were noted in about 24 (14.91%) samples. The most common isolated organism was *Enterococcus faecalis* in 17.3% followed by *Staphylococcus aureus* in 14.29%; this included the three methicillin-resistant S. aureus (MRSA), i.e., 1.86% of the total isolates. There was only one patient in whom Group B Streptococcus (GBS) was isolated.

The antimicrobial sensitivity patterns of the commonly used antibiotics in most frequently occurring organisms are demonstrated in Tables 3-4. The organisms reported in less than or equal to two patients have not been included in the table. The most common organism *E. faecalis* is most sensitive to ampicillin 96.43%, followed by linezolid 89.29% + 3.57% (intermediate sensitivity), vancomycin 85.71% + 10.71% (intermediate sensitivity), and erythromycin being the least sensitive, i.e., 25% + 17.86% (intermediate sensitivity).

**Table 3:** Antimicrobial sensitivity patterns noted in the isolated organisms (in decreasing order of frequency of occurrence) Organisms occurring in ≤ 2 patients have not been taken into the table for the comparison of the antimicrobial sensitivity patterns, I indicates Intermediate sensitivity to the drug. *E. faecalis*: *Enterococcus faecalis*; *E. coli*: *Escherichia coli*; *S. aureus*: *Staphylococcus aureus*; MRSA: methicillin-resistant *Staphylococcus aureus*; *S. epidermis*: *Staphylococcus epidermis*; *S. haemolyticus*: *Staphylococcus haemolyticus*; *C. albicans*: *Candida albicans*; *K. pneumoniae*: *Klebsiella pneumoniae*; *P. aeruginosa*: *Pseudomonas aeruginosa*; *A. baumannii*: *Acinetobacter baumannii*

Antimicrobials	E. faecalis	S. aureus	E. coli	S. haemolyticus	C. albicans	K. pneumoniae	P. aeruginosa	*Enterococcus *spp.	A. baumannii	S. epidermis	*S. aureus *MRSA
Cefepime	NA	NA	83.33%	NA	NA	50%	85.71%	NA	85.71%	NA	NA
Cefuroxime	NA	10%	22.22%	NA	NA	37.00%	NA	14.29%	NA	NA	33.33%
Ampicillin	96.43%	15%	16.67%	0	NA	0	NA	42.86%	28.57%	0	0
Ceftriaxone	NA	15%	27.78%	NA	NA	62.50%	28.57% (I)	14.29%	28.87%	NA	66.67%
Cefoperazone and sulbactam	NA	NA	88.89%	NA	NA	62.50%	42.86%	NA	85.70%	NA	NA
Amikacin	NA	15%	94.44%	NA	NA	75%	85.71% + 14.29% (I)	71.43%	100%	NA	66.67%
Amoxicillin and clavulanic acid	NA	5%	55.56% +5.56% (I)	NA	NA	50%	NA	85.71	14.29%	NA	33.33%
Linezolid	89.29% + 3.57%(I)	85%	NA	100%	NA	12.50%	NA	100%	NA	100%	100%
Erythromycin	25% + 17.86%(I)	10%	NA	10%	NA	NA	NA	14.29%	NA	0%	0%
Gentamicin	7.14%	75%	61.11%	80%	NA	50%	85.71%	85.71%	57.14%	100%	100%
Clindamycin	NA	25%	NA	30%	NA	NA	NA	NA	NA	40%	33.33%
Vancomycin	85.71% + 10.71% (I)	55%	NA	100%	NA	NA	NA	85.71%	NA	80%	33.33%
Piperacillin/tazobactam	NA	NA	88.89%	NA	NA	62.50%	85.71%	NA	57.14%	NA	NA

**Table 4:** Resistance patterns observed in the commonly isolated organisms *E. faecalis*: *Enterococcus faecalis*; *E. coli*: *Escherichia coli*; *S. aureus*: *Staphylococcus aureus*; MRSA: methicillin-resistant *Staphylococcus aureus*; *S. epidermis*: *Staphylococcus epidermis*; *S. haemolyticus*: *Staphylococcus haemolyticus*; *C. albicans*: *Candida albicans*; *K. pneumoniae*: *Klebsiella pneumoniae*; *P. aeruginosa*: *Pseudomonas aeruginosa*; *A. baumannii*: *Acinetobacter baumannii*

Antimicrobials	E. faecalis	S. aureus	E. coli	S. haemolyticus	C. albicans	K. pneumoniae	P. aeruginosa	*Enterococcus *spp.	A. baumannii	S. epidermis	*S. aureus *MRSA
Cefepime	NA	NA	11.11%	NA	NA	25%	14.29%	NA	14.29%	NA	NA
Cefuroxime	NA	5%	66.67%	NA	NA	62.50%	14.29%	71.43%	14.29%	NA	33.33%
Ampicillin	0	85%	61.11%	100%	NA	100.00%	14.29%	57.14%	0	100%	100%
Ceftriaxone	NA	5%	55.56%	NA	NA	37.50%	0	85.71%	0	NA	0
Cefoperazone and sulbactam	NA	NA	5.56%	NA	NA	25%	14.29%	14.29%	14.29%	NA	NA
Amikacin	NA	NA	5.56%	NA	NA	25%	0	28.57%	0%	NA	0
Amoxicillin and clavulanic Acid	NA	10%	22.22%	10%	NA	37.50%	NA	14.29%	0	NA	33.33%
Linezolid	7.14%	5%	NA	0%	NA	0%	NA	0%	NA	0%	0%
Erythromycin	57.14%	70%	NA	90%	NA	NA	NA	0%	0%	100%	100%
Gentamicin	3.57%	15%	22.22%	20%	NA	25%	14.29%	14.29%	0%	0%	0%
Clindamycin	NA	45%	5.56%	50%	NA	NA	NA	NA	NA	40%	0%
Vancomycin	3.57%	25%	NA	0%	NA	NA	NA	0%	NA	20%	0%
Piperacillin/tazobactam	NA	NA	5.56%	NA	NA	12.50%	0%	NA	14.29%	NA	NA

The second most common organism, i.e., *S. aureus* was found to be most sensitive to linezolid 85% followed by gentamicin 75% and vancomycin 55%; MRSA detected in three patients was found to have 100% sensitivity to gentamicin and linezolid, 66.67% sensitivity to ceftriaxone and amikacin, and 33.33% sensitivity to cefuroxime and amoxicillin and clavulanic acid.

*Escherichia coli*, the third commonest, was found to be most sensitive to amikacin 94.44% followed by cefoperazone and sulbactam and piperacillin/tazobactam both with 88.89% sensitivity each; followed by cefepime 83.33%. Which was followed by gentamicin 61.11%, amoxicillin and clavulanic acid 55.56% + 5.56% (intermediate sensitivity).

*Staphylococcus haemolyticus* was found to be 100% sensitive to linezolid and vancomycin followed by 80% of them being sensitive to gentamicin, 30% sensitivity was observed to clindamycin, and 0% sensitivity to ampicillin.

*Klebsiella pneumoniae* was found to have 75% sensitivity to amikacin, 62.5% sensitivity to ceftriaxone, cefoperazone and sulbactam, and piperacillin/tazobactam. 50% sensitivity was noted to cefepime, gentamicin, and amoxicillin and clavulanic acid.

The one patient in which GBS was detected was found to be sensitive to cefuroxime, ampicillin, ceftriaxone, cefoperazone and sulbactam, amoxicillin and clavulanic acid, erythromycin, clindamycin, and intermediate sensitivity to gentamicin.

**Figure 1:**
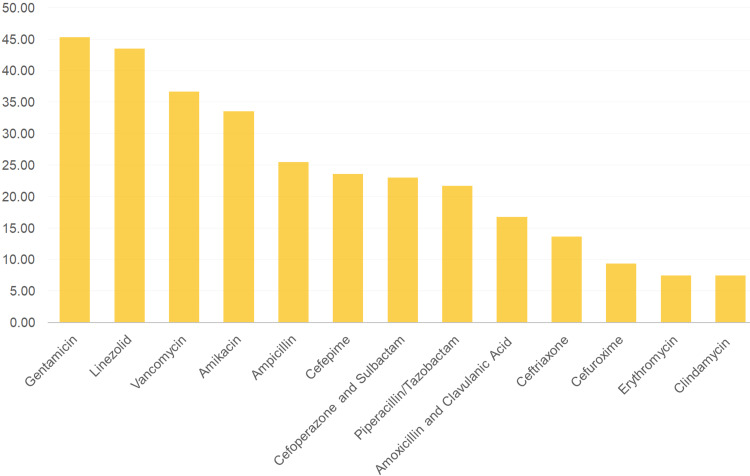
Decreasing trend of the sensitivity pattern of antibiotics as detected in the high vaginal swab culture

Figure 1 depicts the pattern of the most sensitive to the least sensitive to antibiotic pattern detected in this study. As observed, gentamicin is found the most sensitive, followed by linezolid, vancomycin, amikacin, and ampicillin. Less than 50% sensitivity was observed in decreasing order of the antibiotics mentioned as cefepime, cefoperazone and sulbactam, piperacillin/tazobactam, and amoxicillin and clavulanic acid. Less than 30% sensitivity was noted in ceftriaxone, cefuroxime, erythromycin, and clindamycin. No sensitivity was detected in cefotaxime and metronidazole.

## Discussion

Vaginal infection has been associated with increased risks for preterm labor and PROM. As reported in studies conducted earlier, *Ureaplasma urealyticum*, Sneathia species, *E. coli*, *Chlamydia trachomatis*, *Mycoplasma hominis*, *E. faecalis*, and *Gardnerella vaginalis* are the common bacteria associated with PPROM [6,7]. In our study, the most common organism is the *E. faecalis* as detected in 17% of samples, whereas Enterococcus spp. was observed in 4.35% of the total cultures. *S. aureus* was observed in 14.29%; of which 1.86% was MRSA followed by *E. coli* as detected in 11.18% of the cultures. No growth was seen in 14.91%. The reason for the change in the spectrum of vaginal flora might be the geographical factors, routine behavioral changes like perineal hygiene, use of chemicals, soaps, cosmetics, and use of over-the-counter antibiotics. As there is a lack of data on the total profile of genital tract bacteria among Indian women with PPROM and considering the varied diversity of the organisms as seen in our study, i.e., within the same resource setting, multicentric studies need to be done.

In a study conducted by Singh et al. in 2016, PPROM causative agents were *E. coli* in 29.5%, followed by Enterococci (19.6%), Candida (17.6%), *G. vaginalis* (11.7%), and no GBS were reported [8]. In their antibiogram study, *E. coli* was highly sensitive to linezolid and vancomycin followed by amikacin (90%), cefoperazone, sulbactam, and nitrofurantoin (80%) whereas resistance to cefotaxime (80%), amoxicillin and clavulanic acid (70%) was noted. *S. aureus* was more sensitive to linezolid, vancomycin (100%) followed by cefotaxime 90%, clindamycin 80%, nitrofurantoin 60%, and mostly resistance to amoxicillin and clavulanic acid. Enterococcus species was mostly sensitive to vancomycin 100%; amoxicillin and clavulanic acid, amikacin 90%, and it is highly resistant to ampicillin. Except for low incidence of GBS detected in both studies, the present study is not consistent with their study. This discrepancy might be due to the exclusion criteria where they have not excluded those who used antibiotics before the clinical presentation and leaking per vagina > 24 hours.

A study conducted in Chandigarh by Rani et al. reported that the pathogens in PPROM predominantly consist of *E. coli* and *S. aureus* and most of the pathogens were sensitive to gentamicin and ampicillin [9]. Rani et al.’s study is comparable to our study where we found *E. faecalis* followed by *S. aureus* as the most common isolates. In the sensitivity test, most of the organisms are sensitive to gentamicin followed by linezolid instead of ampicillin. A study by Shivaraju et al. conducted in Andhra Pradesh reported that the most common pathogen involved is coagulase-negative *S. aureus*, followed by *Candida albicans*. It also reported that most pathological isolates were sensitive to ampicillin, cefotaxime, and gentamicin which is partially consistent with our study [10].

Beevi et al. reported that the most common gestational age was under late preterm gestation and the majority being primigravida. Out of the 20% positive cultures in their study, the most common organism was *E. coli* (8.6%) and was sensitive to cefoperazone-sulbactam but resistant to ampicillin [11]. It is comparable to our study, where *E. coli* was the third most common organism and most sensitive to amikacin (94.44%) followed by cefoperazone and sulbactam with piperacillin/tazobactam (88.89%) and maximum resistance was seen with cefuroxime followed by ampicillin.

Multiple studies conducted in India reported low or no incidence of GBS, which is also observed in our study. A study in Hong Kong in 2019 by Li et al. reported Gram-positive bacteria in 18.4%, among which GBS was the most common (14.6%); Gram-negative bacteria in 12.8%, among which most common was *E. coli* (8.0%) with resistance to ampicillin (70.3%) and gentamicin (33.3%) whereas co-amoxiclav (3.6%). Intravenous cefuroxime (14.0%) resistance rates were low [12]. The higher rates of resistance to ampicillin and gentamicin in their study may be due to the varied empirical antibiotic prophylaxis protocol in the area. As per the study of Saghafi et al. in Iran, the most common pathogen was *E. coli*, coagulase-negative Staphylococci, Enterococcus, and Candida with the prevalence of 2.2% GBS [13].

As recommended in National Institute For Health and Care Excellence Guidelines 2015, the antibiotic of choice and optimal duration of treatment are not clear; erythromycin 250 mg four times a day for 10 days or until the woman is in established labor (whichever is sooner). For women with PPROM who cannot tolerate erythromycin or in whom erythromycin is contraindicated, consider oral penicillin for a maximum of 10 days or until the woman is in established labor [14]. This approach although simplistic can lead to inadequate treatment if causative organisms are resistant or have intermediate or low sensitivity to these antibiotics. Also, wide spectrum resistance to the penicillin group of antibiotics has been reported previously from India and other developing countries [15]. The resistance patterns in our study also observed similar findings. In resource-limited settings where microbiological evaluation of amniotic fluid is not feasible, identification of bacteria in high vaginal swab can guide antibiotic therapy in women with PPROM including empirical and specific antibiotics according to the most prevalent microorganism in the vagina. The inappropriate and rampant use of antibiotics for multiple reasons has led to varied resistance patterns in pathogens all over the world. The automated culture used in our study provided rapid results in comparison to the manual cultures. It can aid in species specification of a varied range of aerobic organisms and hence a large number of species were identified in our cultures.

The limitation of our study is the lack of special culture media as required for *U. urealyticum*, *C. trachomatis*, Mycoplasma species, and anaerobic organisms in our hospital setup which preclude us to identify any organisms in 24 participants (14.9%).

## Conclusions

Varied diversity of microorganisms and antimicrobial sensitivity was observed in our study population. Predominant organisms in PPROM cases are *E. faecalis* and *S. aureus* with a very low incidence of GBS. The pattern of antimicrobial sensitivity reflected gentamicin being the most sensitive one followed by linezolid, vancomycin, amikacin, and ampicillin. Considering the diversity of the organisms in the vaginal flora, there is a need for further study to see the comparative microbiota in PPROM versus non-PPROM cases. Larger randomized and multicentric studies are needed to formulate the antibiotic policy for PPROM management in women especially in resource-limited settings to avoid further drug resistance to first-line antimicrobials.
